# Impact of Smoking on Household SARS-CoV-2 Transmission

**DOI:** 10.3390/healthcare14040540

**Published:** 2026-02-22

**Authors:** Jèssica Pardos-Plaza, Iván Martínez-Baz, Diana Toledo, Carme Miret, Ignasi Parrón, Joaquim Ferras, Miquel Alsedà, Mònica Carol, Montserrat Zayas, Inma Sanz, Manuel García-Cenoz, Joan A. Caylà, Jesús Castilla, Ángela Domínguez, Pere Godoy, the SARS-CoV-2 Transmission to Household Contacts Working Group (PI21/01883 and ESP22PI01)

**Affiliations:** 1 Escola de Doctorat, Universitat de Lleida, 25003 Lleida, Spain; 2 Institut de Recerca Biomédica (IRB Lleida), 25198 Lleida, Spain; 3 Instituto de Salud Pública de Navarra—IdiSNA, 31008 Pamplona, Spain; 4 CIBER de Epidemiología y Salud Pública (CIBERESP), 28029 Madrid, Spain; 5 Department of Medicine, Universitat de Barcelona, 08036 Barcelona, Spain; 6 Hospital Universitari Arnau de Vilanova, 25198 Lleida, Spain; 7 Agència de Salut Pública de Catalunya, 08005 Barcelona, Spain; 8 Tuberculosis Research Unit Foundation of Barcelona, 08008 Barcelona, Spain

**Keywords:** SARS-CoV-2, smoking, infection, cohabitant, household

## Abstract

**Background/Objectives:** The role of smoking in household SARS-CoV-2 transmission is controversial. The objective of this study was to analyze the impact of smoking on SARS-CoV-2 index cases and transmission to household contacts. **Methods:** Prospective cohort study of 227 index cases and 332 household contacts in Catalonia and Navarre (May 2022–December 2024). The primary outcome measure was SARS-CoV-2 infection in contacts, confirmed by rapid antigen and polymerase chain reaction testing. Adjusted odds ratios (aOR) and 95% confidence intervals (CI) for the smoking-infection association were calculated using logistic regression, adjusting for age, vaccination, and previous infection. **Results**: The cumulative infection incidence in contacts was 38.6%, and was higher in people aged ≥65 years (60.6%) and in smokers (48.6%) vs. non-smokers (33.8%). In the multivariate analysis, index case vaccination (aOR = 0.27; 95%CI: 0.11–0.63) and previous contact infection (aOR = 0.49; 95%CI: 0.30–0.81) were associated with a lower probability of transmission, while smoking by household contacts (aOR = 2.09; 95%CI: 1.19–3.65) and age ≥ 65 years (aOR = 5.13; 95%CI: 2.18–12.09) were associated with an increased risk of infection. The index case smoking status was not statistically significant. **Conclusions:** Smoking by cohabitants and age ≥ 65 years increase the risk of SARS-CoV-2 infection. Index case vaccination and previous contact infection are associated with reduced intra-household transmission.

## 1. Introduction

Smoking is one of the main threats to public health worldwide, with the World Health Organization (WHO) estimating smoking to be responsible for some 7 million deaths annually, 1.6 million corresponding to non-smokers passively exposed to environmental tobacco smoke [1]. In Spain, according to the 2023 National Health Survey, 23.3% of men and 15.5% of women aged over 15 years are declared smokers [2].

A possible association between smoking and COVID-19 has been the subject of scientific attention and controversy, given that SARS-CoV-2 infection primarily affects the respiratory system [3]. Several studies have reported that smoking increases the severity of SARS-CoV-2 infection [4,5,6], while a Spanish 2021 systematic review and meta-analysis concluded that both smokers and ex-smokers were at increased risk of adverse disease progression [5]. In contrast, a French study conducted in patients hospitalized with COVID-19 in Lyon observed that greater COVID-19 severity was associated only with being a former smoker [6]. Other studies have also shown that smokers were more likely to develop severe COVID-19, although the results were not statistically significant [4,7].

A number of studies [8,9,10] report contradictory results regarding the impact of smoking on the risk of SARS-CoV-2 infection. A 2020 Chinese study suggested that nicotine may have modulating effects that reduce infection likelihood and disease severity [8]. The same year, Lippi et al. [9] published a meta-analysis concluding that active smoking was not significantly associated with an increased risk of progression to severe COVID-19. In 2021, after analyzing different studies, Simons et al. [10] concluded that current smokers appeared to have a lower risk of SARS-CoV-2 infection, while former smokers showed a higher risk of hospitalization, severity, and mortality from COVID-19.

Despite the extensive literature on the relationship between smoking and COVID-19, most studies [4,5,6] have focused on clinical severity, whereas the role of smoking in infection risk has been unexplored.

The present study provides prospective evidence on the role of smoking in household transmission, assessing the independent impact of both index cases and household contacts. This approach allows evaluation of whether smoking primarily increases susceptibility to infection among contacts or also influences transmissibility associated with the index case.

The aim of this study of the impact of smoking among COVID-19 index cases and SARS-CoV-2 transmission to their household contacts was to evaluate the likelihood of smokers infecting household contacts and the role of smoking in infection susceptibility.

## 2. Materials and Methods

### 2.1. Study Design

Between May 2022 and December 2024, we conducted a prospective epidemiological cohort study in Catalonia and Navarre (Spain) aimed at analyzing the role played by smoking in COVID-19 index cases and in SARS-CoV-2 infection of their household contacts.

### 2.2. Participants

The study population consisted of confirmed COVID-19 cases (index cases) and their household contacts in Catalonia and Navarre, as registered by epidemiological surveillance services. Index cases and their household contacts were recruited from 8 primary care centres (7 in Catalonia and 1 in Navarre). Participating primary care centres were selected based on convenience criteria established in each region.

Index cases were considered to be cases of SARS-CoV-2 infection confirmed by a rapid antigen test (RAT) and real-time polymerase chain reaction (RT-PCR) test within the 10 days prior to epidemiological surveillance service notification. Index cases were required to have at least one identified household contact. Both index cases and household contacts granted their verbal consent to participate. No age restrictions were applied.

Excluded were individuals with severe cognitive, visual, or hearing impairments that would make interviewing difficult, persons not resident in Catalonia or Navarre, and index cases without household contacts.

Household contacts, defined as having spent at least 2 h with the index case at the same address in the period from 2 days before diagnosis to infection confirmation, were followed up for 7 days to assess possible infection transmission. All contacts underwent a RAT on day 0, and those who tested negative underwent RT-PCR testing after 7 days. The 7-day follow-up period was established in accordance with the contact tracing protocol in force at the time of the study, for the early detection of secondary cases in the household setting.

### 2.3. Questionnaire Design

Questionnaires were designed following the WHO, the European Centre for Disease Prevention and Control (ECDC), and the Spanish Ministry of Health COVID-19 recommendations and applying a methodology described elsewhere [11].

The questionnaires collected data on sociodemographics, comorbidities, risk factors (smoking), COVID-19 knowledge, preventive measures, previous SARS-CoV-2 infections, and vaccination status. The collected data were validated by linking electronic medical records with regional vaccination registries and epidemiological surveillance databases.

The variable “previous SARS-CoV-2 infection” was defined as the presence of at least one prior episode of COVID-19 confirmed by diagnostic testing and recorded in epidemiological surveillance systems and electronic clinical records before the participant’s inclusion in the study. This variable was considered dichotomously (yes/no), and no specific minimum retrospective period was established in relation to the index case episode.

### 2.4. Data Collection

The study data were collected in questionnaires completed by the project’s research staff in telephone interviews with the index cases and their household contacts. The following data were collected: demographic variables (age and sex); date of onset of first symptoms; specific symptoms; diagnostic tests (RAT, RT-PCR); exposure time to the index case; history of SARS-CoV-2 infection; and risk factors (comorbidities and smoking).

Smoking history was categorized as follows: daily smoker, sporadic smoker (once a week), ex-smoker (since ≥6 months previously), and never-smoker. To enhance statistical robustness, smokers and ex-smokers were grouped together. After 30 days, the clinical evolution of the index cases and their household contacts was reviewed for complications (pneumonia, hospital admission, intensive care unit (ICU) admission, days admitted, and death).

### 2.5. Sample Size

A total of 227 index cases and 332 household contacts participated in the study. This sample size enabled the prevalence of smokers to be estimated as precision (*e*) with a confidence interval (CI) of 95%. For household contacts, with *e* = ±3%, and for index cases, with *e* = ±4%, the calculation formulae were as follows:

e = √(Zα^2^ × p × (1 − p)/n) e = √(1.96 × 0.2 × (0.8)/332 **e** = **0.03**

e = √(Zα^2^ × p × (1 − p)/n) e = √(1.96 × 0.4 × (0.6)/227 **e** = **0.04**

### 2.6. Statistical Analysis

The statistical relationship between the dependent variable, SARS-CoV-2 infection in household contacts, and the main independent variable, smoking history, was assessed using the chi-square test or student *t*-test, for a significance level of *p* < 0.05. Using logistic regression models, the association between smoking history and SARS-CoV-2 infection in household contacts was assessed using the odds ratio (OR) and its 95% CI. Models were adjusted for age (0–17, 18–44, 45–64, and ≥65 years), sex, prior household contact infection, and smoking history and COVID-19 vaccination status of both index cases and household contacts using the backward selection method, retaining in the final model the smoking history and COVID-19 vaccination status and the variables with *p* < 0.10.

Data were analyzed using the EpiInfo v. 7.2.5 software package.

### 2.7. Ethics

This study was approved by the Ethics Committee of the Arnau de Vilanova University Hospital (code: CEIC-2464) and was carried out in accordance with the principles of the Declaration of Helsinki. All subjects included in the study received detailed information on the study aims and granted their consent to participate.

### 2.8. Artificial Intelligence Involvement

The authors declare that no artificial intelligence tools were used in the preparation of this manuscript.

## 3. Results

The sample included 227 COVID-19 index cases and 332 household contacts with a cumulative incidence of infection of 38.6%. Cumulative incidence was highest in contacts aged ≥65 years (60.6%), followed by the 18–44 (38.9%) and 45–64 (34.8%) age groups, and was lowest in the 0–17 age group (23%) (Table 1). Cumulative incidence was higher for contacts with, compared to contacts without, a smoking history (48.6% vs. 33.8%; *p* = 0.009). No significant differences were observed in relation to the smoking history of the index case (35.1% vs. 40.8%; *p* = 0.298) (Table 2).

In the analysis of factors associated with SARS-CoV-2 infection of household contacts (Table 2), a significant association was observed with age: the probability of infection was significantly higher in contacts aged 18–44 and ≥65 years than contacts aged 0–17 years (OR = 2.13; 95%CI: 1.04–4.38 and OR = 5.15; 95%CI: 2.50–10.6 respectively). Smoking by contacts was also associated with an increased risk of infection (OR = 1.85; 95%CI: 1.16–3.00). The probability of re-infection was lower in contacts with a previous history of SARS-CoV-2 infection (OR = 0.48; 95%CI: 0.31–0.75). Previous index case vaccination was associated with a significant reduction in household transmission (OR = 0.30; 95%CI: 0.13–0.63). No statistically significant associations with infection risk were observed for household contact sex, household contact vaccination history, or index case smoking status.

In the logistic regression model (Table 3), after adjusting for age, sex, previous infection, and vaccination against COVID-19, household contacts aged ≥65 years ran a significantly higher risk of infection compared to the 0–17 age group (adjusted OR = 5.13; 95%CI: 2.18–12.09), and smoking in contacts was shown to be a risk factor (adjusted OR = 2.09; 95%CI: 1.19–3.65). In contrast, a previous SARS-CoV-2 infection was significantly associated with a lower likelihood of infection by the index case (adjusted OR = 0.49; 95%CI: 0.30–0.81). Vaccination of the index case continued to show a protective effect against household transmission (adjusted OR = 0.27; 95%CI: 0.11–0.63). Other variables, such as the household contact’s vaccination history and the index case’s smoking status, were not statistically significant in the adjusted logistic regression model.

## 4. Discussion

We analyzed COVID-19 index cases and their household contacts to determine the likelihood of smokers infecting household contacts and the role of smoking in infection susceptibility. Adjusted logistic regression analysis revealed that a smoking history in household contacts doubled their risk of SARS-CoV-2 infection. However, a smoking history in index cases did not increase the risk of transmission to household contacts. The absence of a statistically significant association between the smoking status of the index case and household transmission should be interpreted with caution, as the study may have had limited statistical power to detect effects of small magnitude.

A significant association was found between the probability of infection and age, with contacts aged ≥65 years significantly more likely to be infected than younger age groups. These results are consistent with previous studies reporting intra-household SARS-CoV-2 transmission rates of 11–30%, and higher rates in adults aged ≥65 years, when the index case presented symptoms, index case isolation was delayed, or preventive measures were defective [12,13,14,15]. Immunosenescence and comorbidities such as diabetes make persons aged ≥65 years more susceptible to SARS-CoV-2 infection. The fact that they may also need assistance with activities of daily living implies closer physical contact with family members.

Direct injury to the respiratory immune system caused by smoking may explain the greater susceptibility to infection of household contacts and may even lead to more severe forms of disease, as has been observed for influenza [16]. However, we did not observe a significant association for infection transmission to household contacts by index cases with a smoking history. This result contradicts a finding for tuberculosis that index case smoking was associated with a higher prevalence of tuberculosis infection in index case contacts [17]. This contradiction is possibly explained by the fact that, while both diseases share the same respiratory route, they differ in the types of lesions that facilitate transmission and the contagion periods. Furthermore, it is important to note that, in the case of COVID-19, other factors, such as respiratory viral load and hygiene measures, also influence viral transmission [18]. These findings support the hypothesis that smoking primarily increases host susceptibility to infection rather than viral transmissibility within the household setting.

Although some early studies hypothesized a possible protective effect of nicotine through its interaction with ACE2 receptors, the available evidence indicates that any such theoretical effect is largely outweighed by the structural and immunological damage associated with smoking [16,17]. Alterations in respiratory defence mechanisms and immune response may explain the greater susceptibility to infection observed in smokers, in consonance with our results and with previous COVID-19 studies [3,4,5,6].

During the 30-day follow-up, a limited number of severe clinical events were observed. However, the study lacked statistical power to assess the association between smoking and severity. Smoking, in addition to facilitating infection, may also increase the severity of COVID-19 cases. Several systematic reviews and meta-analyses have shown that smokers compared to non-smokers run higher risks of severe illness, ICU admission, and mortality [3,5]. Cohort studies confirming this association indicate that smoking is associated with a more unfavourable clinical evolution [4,6]. Furthermore, recent research by Bowsher et al. [19] suggests that smoking and vaping modify the genetic expression of several genes related to the susceptibility and severity of SARS-CoV-2 infection, and possibly increasing vulnerability to infection.

Smoking increases susceptibility to both viral and bacterial infections. Studies have shown that smokers are more likely to develop both respiratory infections (common cold, influenza, pneumonia, tuberculosis) [16,17] and non-respiratory infections (meningococcal meningitis, invasive bacterial pneumococcal infections, post-surgical infections and septic complications [20]). Smoking also alters respiratory-tree defence mechanisms (impairing mucociliary and epithelial functioning) and compromises innate and adaptive immunity (macrophages, T and B lymphocytes [20]). Smoking is also associated with multiple infections with a more severe clinical course and greater mortality [21]. Diseases such as pneumonia affect smokers more in terms of greater frequency, severity, mortality, and poorer response to treatment [20].

Our results show that vaccination of index cases and previous infection in household contacts acted as protective factors against intra-household transmission, corroborating studies that demonstrate the impact of acquired immunity on reducing secondary transmission [11,22].

Our findings overall point to the importance of implementing various public health actions and strategies. A combination of preventive population-targeted measures such as vaccination, anti-smoking campaigns, and hygiene education can help reduce the transmission of SARS-CoV-2 and other respiratory infections in the home setting. Preventive measures, such as good hygiene practices (handwashing, home ventilation, and other non-pharmacological measures) are especially important for persons aged ≥65 years, and the risk of infection between smoking cohabitants highlights the need for smoking cessation programmes and health education, especially bearing in mind that, in the Spanish population, the prevalence of smoking continues to be very high, at 25.8% [2,23].

According to our results, smoking status should be systematically recorded and taken into account in the risk assessment of household contacts, particularly in households with older or vulnerable individuals, in order to prioritize and implement preventive interventions.

### Strengths and Limitations

Our study has some limitations that should be taken into account. First, the selection of primary care centres was based on convenience sampling, which may limit the generalizability of the results. Nevertheless, the inclusion of participants from two different autonomous communities and the use of an identical follow-up protocol strengthen the internal consistency of the findings.

Second, the number of cigarettes smoked was not recorded, and in order to ensure the statistical power of the models, current smokers and former smokers were grouped into a single exposure category. This methodological decision may have diluted potential differences associated with smoking intensity or the time elapsed since smoking cessation and may have underestimated or overestimated the true association between smoking and infection risk.

Smoking history is self-reported, so respondents may have responded according to what they consider most socially acceptable, and, given the negative view of smoking, some responses may underestimate exposure. Since the sample size was small, the smoker and ex-smoker categories were combined for reasons of statistical robustness. The impact of smoking may also vary, depending on the number of cigarettes smoked and the number of years’ smoking, and such data were not included in this study. Since vaccinated individuals and older individuals may have fewer symptoms, infections may have gone undetected; however, we consider this to be unlikely, as all contacts who tested negative on day 0 were retested after 7 days, regardless of whether or not they had symptoms. Finally, since the study was observational, causality could not be directly linked to smoking. In addition, although the analysis was conducted at the individual level, some degree of non-independence between observations within the same household cannot be completely ruled out, which may have affected the precision of the estimates.

The follow-up, limited to 7 days, may have underestimated the detection of infections with longer incubation periods and therefore, it cannot be ruled out that some secondary cases may have gone undetected. In addition, as no specific time window was defined for previous SARS-CoV-2 infection, it was not possible to assess whether the observed protective effect depends on the time elapsed since the infection.

Furthermore, the possible presence of unmeasured comorbidities, particularly among older participants, may have influenced susceptibility to infection.

On the other hand, no detailed information was collected on behaviours associated with tobacco use, such as smoking in outdoor spaces or maintaining distance from other household members, which could have acted as unmeasured confounding factors.

Regarding study strengths, its prospective design enabled a temporal relationship to be established between smoking and SARS-CoV-2 infection. Recruitment was based on a contact tracing protocol applied to the entire population and that remained unchanged throughout the study period. Participant variables were collected before test results were known, and contacts were classified based on their results.

Our findings have important public health implications in that they highlight the need for smoking cessation programmes, preventive measures (especially for those aged ≥65 years), and the importance of vaccination.

Although scientific evidence exists on the relationship between smoking and COVID-19 [7], most studies have focused on clinical severity [4,5,6]. In contrast, the impact of smoking on SARS-CoV-2 infection risk has been less studied, and data on its influence on intra-household transmission remain scant.

## 5. Conclusions

In our study, smoking by household contacts was associated with an increased risk of infection regardless of whether the index case smoked or not. Vaccination of index cases and a history of previous infection in household contacts acted as protective factors. Our findings reinforce the importance of promoting smoking cessation, maintaining high vaccination coverage, and strengthening preventive measures for all smokers.

Future studies should investigate this association by differentiating between current and former smokers and incorporating more detailed measures of tobacco exposure.

Similarly, studies with larger sample sizes should explore the possible interaction between smoking and older age, taking into account immunosenescence processes.

## Figures and Tables

**Table 1:** Cumulative SARS-CoV-2 infection incidence in household contacts of COVID-19 index cases.

Variable	Infected Contacts n = 128 (%)	Total Contacts n = 332
**Age (years)**		
0–17	17 (23.0)	74
18–44	28 (38.9)	72
45–64	40 (34.8)	115
≥65	43 (60.6)	71
**Sex**		
Female	67 (42.1)	159
Male	61 (35.3)	173
**HOUSEHOLD CONTACTS**		
**Smoker**		
Yes	52 (48.6)	107
No	76 (33.8)	225
**Vaccination ≥ 1 dose**		
Yes	117 (38.4)	305
No	11 (40.7)	27
**Previous COVID-19**		
Yes	54 (30.5)	177
No	74 (47.7)	155
**INDEX CASES**		
**Vaccination**		
Yes	107 (35.7)	300
No	21 (65.6)	32
**Smoker**		
Yes	46 (35.1)	131
No	82 (40.8)	201

**Table 2:** Factors associated with cumulative SARS-CoV-2 transmission from COVID-19 index cases to their household contacts at the bivariate level.

Variable	Contacts		OR	95% CI	*p* Value
	Infected n = 128	Non-Infected n = 204			
Age (years) ± SD	44.2 (23.16)				
**Age (years)**					
0–17	17 (22.97)	57 (77.03)	Ref.		
18–44	28 (38.89)	44 (61.11)	2.13	1.04–4.38	0.04
45–64	40 (34.78)	75 (65.22)	1.79	0.92–3.5	0.09
≥65	43 (60.56)	28 (39.44)	5.15	2.50–10.6	0.001
**Sex**					
Male	61 (35.26)	112 (64.74)	0.75	0.48–1.16	0.20
Female	67 (42.14)	92 (57.86)	Ref.		
**HOUSEHOLD CONTACTS**					
**Smoker**					
Yes	52 (48.60)	55 (51.40)	1.85	1.16–3.00	0.009
No	76 (33.78)	149 (66.22)	Ref.		
**Vaccination ≥ 1 dose**					
Yes	117 (38.36)	188 (61.64)	0.90	0.41–2.02	0.807
No	11 (40.74)	16 (59.26)	Ref.		
**Previous COVID-19**					
Yes	54 (30.51)	123 (69.49)	0.48	0.31–0.75	0.001
No	74 (47.74)	81 (52.26)	Ref.		
**INDEX CASES**					
**Vaccination**					
Yes	107 (35.67)	193 (64.33)	0.3	0.13–0.63	0.001
No	21 (65.63)	11 (34.38)	Ref.		
**Smoker**					
Yes	46 (35.11)	85 (64.89)	0.8	0.49–1.24	0.298
No	82 (40.80)	119 (59.20)	Ref.		

CI: confidence interval; OR: odds ratio; SD: standard deviation.

**Table 3:** Factors associated with cumulative SARS-CoV-2 transmission from COVID-19 index cases to their household contacts at multivariate level.

Variable	aOR	95% CI	*p* Value
**Age (years)**			
0–17	Ref.		
18–44	2.23	0.93–5.40	0.074
45–64	1.71	0.74–3.95	0.207
≥65	5.13	2.18–12.09	0.001
**Sex**			
Male	0.64	0.39–1.05	0.078
Female	Ref.		
**HOUSEHOLD CONTACTS**			
**Smoker**			
Yes	2.09	1.19–3.65	0.009
No	Ref.		
**Vaccination ≥ 1 dose**			
Yes	0.52	0.18–1.49	0.218
No	Ref.		
**Previous COVID-19**			
Yes	0.49	0.30–0.81	0.005
No	Ref.		
**INDEX CASES**			
**Vaccination**			
Yes	0.27	0.11–0.63	0.003
No	Ref.		
**Smoker**			
Yes	0.80	0.48–1.33	0.384
No	Ref.		

aOR: adjusted odds ratio (according to the remaining variables in the table); CI: confidence interval.

## Data Availability

The data used for this study are available on request to the corresponding author.

## Abbreviations

aOR	adjusted odds ratio
CI	confidence interval
COVID-19	coronavirus disease 2019
OR	odds ratio
RAT	rapid antigen test
RT-PCR	real time polymerase chain reaction
SARS-CoV-2	severe acute respiratory syndrome coronavirus 2
SD	standard deviation
